# A Cascaded Multi-Agent Reinforcement Learning-Based Resource Allocation for Cellular-V2X Vehicular Platooning Networks

**DOI:** 10.3390/s24175658

**Published:** 2024-08-30

**Authors:** Iswarya Narayanasamy, Venkateswari Rajamanickam

**Affiliations:** Department of Electronics and Communication Engineering, PSG College of Technology, Coimbatore 641004, India; rvi.ece@psgtech.ac.in

**Keywords:** radio resource management, vehicle platooning, Age of Information, Multi-Access Edge Computing, Multi-Agent Reinforcement Learning

## Abstract

The platooning of cars and trucks is a pertinent approach for autonomous driving due to the effective utilization of roadways. The decreased gas consumption levels are an added merit owing to sustainability. Conventional platooning depended on Dedicated Short-Range Communication (DSRC)-based vehicle-to-vehicle communications. The computations were executed by the platoon members with their constrained capabilities. The advent of 5G has favored Intelligent Transportation Systems (ITS) to adopt Multi-access Edge Computing (MEC) in platooning paradigms by offloading the computational tasks to the edge server. In this research, vital parameters in vehicular platooning systems, viz. latency-sensitive radio resource management schemes, and Age of Information (AoI) are investigated. In addition, the delivery rates of Cooperative Awareness Messages (CAM) that ensure expeditious reception of safety-critical messages at the roadside units (RSU) are also examined. However, for latency-sensitive applications like vehicular networks, it is essential to address multiple and correlated objectives. To solve such objectives effectively and simultaneously, the Multi-Agent Deep Deterministic Policy Gradient (MADDPG) framework necessitates a better and more sophisticated model to enhance its ability. In this paper, a novel Cascaded MADDPG framework, CMADDPG, is proposed to train cascaded target critics, which aims at achieving expected rewards through the collaborative conduct of agents. The estimation bias phenomenon, which hinders a system’s overall performance, is vividly circumvented in this cascaded algorithm. Eventually, experimental analysis also demonstrates the potential of the proposed algorithm by evaluating the convergence factor, which stabilizes quickly with minimum distortions, and reliable CAM message dissemination with 99% probability. The average AoI quantity is maintained within the 5–10 ms range, guaranteeing better QoS. This technique has proven its robustness in decentralized resource allocation against channel uncertainties caused by higher mobility in the environment. Most importantly, the performance of the proposed algorithm remains unaffected by increasing platoon size and leading channel uncertainties.

## 1. Introduction

Ultra-Reliable Low-Latency Communication (URLLC) is one among the significant vertices of the 5G triangle. The 3rd Generation Partnership Project (3GPP) describes URLLC’s reliability, assuring 99.99% for transmission of a 32-byte data frame and an E2E latency of 1 ms for both uplink and downlink. URLLC holds certainty in mission-critical applications such as industrial automation, the healthcare industry, intelligent transportation, media, smart electricity grid, and so on. Accounting for one of the mission-critical applications of Intelligent Transportation Systems (ITSs), transportation efficiency and safety have emerged as a serious predicament—as indicated by the World Health Organization (WHO) in the Global Status Report on Road Safety [1] about the increased road accident rates—and also alarm us regarding the huge growth in the number of vehicles to 2 billion by the year 2050.

Our research primarily focuses on Intelligent Transportation Systems (ITSs) that have become absolutely futuristic for traffic management, facilitating users with extended safety, coordination, and smartness [2]. To specify, Tesla’s Autopilot, Google’s Waymo, and Baidu’s Apolong are eminently fascinating in driverless technology, reckoning one-fourth of prospectus vehicles are expected to be autonomous in 2030. The onset of the IEEE 802.11p Vehicle-to-Everything (V2X) communication standard that uses WLAN technology appears to be a pivotal innovation to guarantee safety, energy savings, and an effective ITS. This technology communicates in two modes: Vehicle-to-Infrastructure (V2I) and Vehicle-to-Vehicle (V2V). The former one, V2I, is responsible for high data rate messages between vehicles and infrastructure, which are Road Side Units (RSU). On the contrary, V2V communication focuses on delivering safety-critical messages between connected vehicles [3]. Through systemized data exchange, latency in the vehicle’s response time is reduced by avoiding eventual hurdles.

The performance of Long-Term Evolution (LTE) for V2X communications in the Third Generation Partnership Project (3GPP) has been analyzed [4,5], in which eNodeBs are responsible for handling Radio Resource Management (RRM). However, the traditional LTE architecture could not achieve the demands of Quality of Service (QoS) required for V2X applications.

Consequently, Device-to-Device (D2D) communication emerged as the ongoing technology for prospective cellular systems, especially in meeting the needs of safety-critical V2X applications. In D2D communication, devices that are geographically close to each other communicate by sharing the same resources just like Cellular User Equipment (C-UE), with the possibilities of high reliability through proximity gain, low latency through hop gain, and spectrum utilization through reuse gain [6]. Meanwhile, reusing the same resources drives interference, and accomplishing the necessary latency and reliability in V2X communications becomes cumbersome. Eventually, RRM is focused on as a crucial problem encountered by the communications in V2X applications.

Simultaneously, ITS fosters autonomous vehicular platooning. The platooning maneuver is a promising technology for autonomous vehicles, particularly for handling traffic and prevailing transportation costs. Moreover, another major hindrance in the urban road system is intersection capacity utilization, which is just a fragment of actual intersection capacity. As an alternative to the ensuing of vehicles, platooning the vehicles strengthens the intersection capacity three-fold [7]. In brief, platooning refers to a convoy of interconnected cars or trucks that coordinate their kinetics and mobility pattern regularly. In each platoon formed, there will be a lead or Captain Vehicle (CV) and a group of Follower Vehicles (FV). The Captain Vehicle is responsible for communication with corresponding platoon vehicles, and the Follower Vehicles trail one another closely [8].

In a platooning system, critical conditions to be examined and ensured are string stability and stringent latency in the broadcasting of messages. The stability of a string of vehicles is defined as, for any set of bounded initial disturbances to all the vehicles, the position fluctuations of all the vehicles remain bounded and these fluctuations approach zero as time t →∞ [9]. The other condition involves the broadcasting of messages like the acceleration, velocity, location, and position of vehicles, which comprise the Cooperative Awareness Messages (CAM) in ETSI or Basic Safety Message (BSM) in SAE standards that are frequently established by the CV to organize the platoon [10].

Furthermore, a vast amount of data are extracted by autonomous vehicles, and the computing ability required to manage these extracted data is lacking. To tackle the aforesaid problem, state-of-the-art data processing technology cited as Cloud Computing (CC) technology is concerned. Nonetheless, in conventional cloud computing, information dissemination between the far-placed vehicles and the remote cloud leads to an extreme delay, thereupon causing incompatible mobility scenarios. By placing an MEC server at the LTE Base Station (BS), storage and computing resources can be provided, which in turn enhances the network capability. Hence, Multi-access Edge Computing (MEC) is appraised as a promising solution to facilitate computational resources for Automated Vehicles (AVs) to minimize latency.

Meanwhile, the conventional way of optimizing no longer serves the significant growth of AVs owing to the uncertain constraints analogous to heightened complexities and a weak convergence rate. Such identified challenges demand efficient optimization techniques to address the intricate constraints effectively. Reinforcement learning counts as one of the dominant machine-learning tools wherein MADDPG or Multi-Agent Deep Deterministic Policy Gradient is latterly adopted. MADDPG, an expansion of DDPG, comprises the Multi-Agent Policy Gradient, which has been regarded as a potential solution for optimizing radio resource management in AV platooning scenarios.

However, the Q-function employed by MADDPG for discovering the optimum actions by the agent adversely leads to function approximation issues. Consequently, the errors contribute to the overestimated values and, in turn, link to suboptimal policies. Overestimation bias remains prevalent in the actor–critic environment. Hence, novel approaches are necessitated to mitigate the impact on both environments. Therefore, a cascaded approach for optimizing radio resource management in the platooning scenarios by mitigating the estimation bias issues and minimizing the latency is proposed.

### 1.1. Related Works

Vehicle platooning is a salient application of ITS with its promising ability to improve road safety whilst decreasing the fuel consumption of vehicles. Platooning comprises a group of vehicles driving together and simultaneously exchanging information. An overview of distinct projects appearing with vehicle platooning is presented in [11]. The mobility of self-driving cars in the platooning concept is explored. Adaptive cruise control in autonomous vehicles provides a vehicle with the potential to follow another vehicle ahead at entailed intervals to form and maintain the platoons. A string comprising ten vehicles is controlled through several control algorithms involving feedback and feedforward control schemes being applied [12].

Besides conventional centralized–platoon formation algorithms, adopting effective and efficient distributed or decentralized algorithms for platoon formation is recommended [13]. The strategies to validate the existing platooning algorithms are featured in the research review, and a comparative analysis accounting for certain key factors is employed [14]. Although platooning of vehicles has abundant advantages, communication range boundaries are a concern when long platoons of heavy-duty vehicles are formed. The virtual leader concept is proposed, and effective leaders are elected based on VLQI metrics. Hence, L-Platooning is proposed for forming and handling long platoons seamlessly [15].

The complexity entangled in the analysis of platooning has initiated simulative analysis, which is intended to surpass the real-time test-bed constraints. In the aspect of vehicular network and vehicle dynamics, platooning of vehicles is simulated and its potential is showcased [16].

A significant aspect of platooning that enables ITS is its capability to dynamically control the velocity and stability of vehicles traveling in the same platoon. The authors recommend having the control function of platoons extremely near to the platoon; whereas, as previously discussed, CC is inapplicable for delay-critical platooning systems. Recently, researchers started preferring MEC as an enabler for platooning systems. The extensive research on computation offloading in MEC-based V2X schemes focuses on offloading computational tasks from vehicles to MEC servers. Subsequently, platooning can be expedited by Multi-access Edge Computing. Building and maintaining a platoon demands the computational offloading and vehicle coordination enabled by MEC [17].

Having demonstrated the viability of platooning in MEC schemes, integrating MEC-directing platoon control in the V2V method is investigated by exploring the Platooning-as-a-Service (PaaS) model [18]. The Cooperative Adaptive Cruise Control (CACC) utilizing V2X communications focuses on safe driving; yet, other critical drawbacks in CACC are addressed through the MEC-based CAD approach [19]. An emerging IoV scenario involves outrageous computational tasks and performing them becomes strenuous. Therefore, V2X technology is utilized in routing computation tasks and developing a balanced optimal offloading scheme [20]. Primarily, the IEEE 802.11 Distributed Coordination Function (DCF) is adopted by vehicles to communicate information to the BS associated with the edge server. Vehicles traveling in various lanes at varying speeds deliver an uncertain amount of information to the BS causing data processing complications. Thus, joint optimization is formulated for MEC-based V2I networks [21].

Correspondingly, growing traffic needs and diversified applications necessitate tremendous processing and radio resources. MEC and D2D play a key role in addressing the mentioned challenges. A multi-objective optimization problem to maximize task completions and minimize energy and cost in D2D-enabled Het-MEC scenarios is reported [22]. Multi-access Edge Computing also plays a significant role in minimizing the computational delay of edge devices in NOMA schemes. The joint optimization of transmission duration and workload offloading allocation between the edge servers achieves minimized computational delay in downlink NOMA [23].

In the past decade, technologies have evolved persistently to attain smartness in vehicles. It has been realized that processing huge amounts of real-time data with ultra-reliability and delay-sensitivity requirements while possessing limited computational ability in vehicles becomes cumbersome. Thereby, offloading the computations and tasks to the edge cloud and vehicular cloudlet appears to be viable.

Simultaneously, Artificial Intelligence (AI) has profoundly revolutionized the living, specifically in ITS. The prominent achievements of AI in vehicular networks owe to the rise of Reinforcement Learning (RL) and its subfield, Deep Reinforcement Learning (DRL). An extensive review of studies that combine RL/DRL strategies to coordinate vehicular networks, particularly focusing on challenges in vehicular telecommunication, is presented in [24]. Besides, considering the bounded potential of edge nodes, cloud-edge services the offloading decisions. Consequently, a multi-update RL algorithm is formulated for decision-making in delay-sensitive applications in [25]. Despite MEC delivering computational capabilities in the near vicinity, modeling the offloading policies is crucial. An optimization problem is formulated for offloading decision-making based on channel characteristics between UE and BS in [26].

Ultimately, Deep Reinforcement Learning algorithms are applied for optimizing the decisions on task-partitioning and offloading resource management scenarios in MEC. The obtained decision variables are handled individually, impacting the system performance. To surmount the issue and jointly optimize the acquired decision variables, the hybrid action space is addressed. A D3PG model is developed to deal with multi-objective optimization along with task processing maximization, including energy and delay minimization, in [27].

Although DRL techniques are employed for resource management in V2X communication, certain vulnerabilities remain unsolved, including quantization errors caused by hybrid action space and high computational complexity by multi-dimensional quantization [28].

The literature considered thus far has examined the computation offloading and resource management in MEC for quasi-static systems; following this, dynamic MEC systems are attempted. A joint optimization algorithm for offloading computations and allocating resources in a rapidly changing MEC demanding heterogeneous services with delay constraints is devised [29].

Furthermore, intelligent resource management schemes are a significant prerequisite in vehicle platooning for communicating CAM messages between vehicles frequently at regular intervals. A joint optimization algorithm for the resource management and control factors of vehicles in the LTE-V2V network is proposed in [30]. The cell border limitations with the LTE mobility system are addressed by employing 3GPP V2X deployment. These feature enhancements of 3GPP NRV2X are incorporated in Rel 16 and Rel 17 [31]. As the deployment of MEC is regarded in a cellular environment, the high mobility of users creates a bottleneck hindering efficiency and reliability. An optimization technique to overcome the migration/handover challenges under distinct circumstances is proposed in [32].

Usually, the data size for each user-requested computational task differs. It is the responsibility of the BS to efficiently regulate the resource allocation strategy; so, an optimization problem based on a multi-stack reinforcement learning algorithm is formulated to minimize the computation and communication delays between each user [33]. The computation efficiency, termed as the trade-off between computational time and consumed energy, is analyzed in a Vehicular Edge Computing scenario, and the MACTER algorithm is proposed to solve the distinct requirements of the mentioned scenario in [34]. In general, vehicular networks are multiple-agent systems possessing collective behavior among the agents, resulting in uncertainties. The incoordination between the agents leads to oscillations in actions and hinders the overall performance. For this reason, multi-agent RL algorithms have evolved. Multi-agent deep Q-learning algorithms are proposed for optimizing offloading and resource management in the MEC network in [35]. Despite the enhanced computational efficiency and reduced communication latency contributions of MEC, its computing resources are restricted. Therefore, vehicles with huge unused resources in urban areas are utilized for offloading computing tasks attempted through vehicular fog-edge computing [36].

In addition, the vehicular environment is highly dynamic; hence, the training and testing phase possesses a deviation in its environment. In these aspects, the recent DRL algorithms create discrepancies implying indecisive behavior in a rapidly changing environment. To circumvent all the mentioned constraints, the Multi-Agent Deep Deterministic Policy Gradient (MADDPG) among MARL schemes is broadly accepted. The centralized training and distributed execution nature of MADDPG means that every agent gathers knowledge about the others in the training phase and implements the actions independently from the gained knowledge. However, complications emanate as the critic’s intervention develops proportionally to the number of agents also accompanied by incoordination among the agents. In rapidly changing dynamic scenarios, infeasibility in allocating unique resources to each vehicle and the implications of spatially distributed vehicles have contributed to the DIRAL mechanism to enhance PRR performance [37]. The conventional optimization techniques fail to satisfy the expectations of a highly scalable network with channel uncertainties and, hence, distributed resource allocation algorithms have manifested [38]. The resource management framework is modeled as a multi-agent system in which the platoon leader acts as an agent and takes action purely based on channel information gathered locally [39].

The platooning of vehicles is a crucial application of URLLC architecture. The aggregation of time-sensitive safety messages and traffic updates is incredibly significant. The timing accuracy of these messages is framed as Age of Information (AoI). The DQN-based dynamic environment learning method is demonstrated to forecast optimal MCW for intelligent vehicular nodes, guaranteeing age fairness in V2I communications [40]. The AoI minimization problem in the AB-EH-CRN Network is handled using the HAEP-DDPG algorithm, particularly evolving to the continuous and discrete action space combinations [41].

The performance optimization of AoI-aware radio resource allocation has been investigated in a Manhattan V2V network recently [42]. However, the proposed scheme focuses on delivering CAM messages with 99% transmission probability by guaranteeing minimized time lapse in the platooning URLLC application.

### 1.2. Contributions and Paper Organization

We focus on AoI minimization optimization in a highly dynamic vehicle platooning. The considered platooning system comprises various autonomous vehicles in which CVs are responsible for communicating between the vehicle pairs and broadcasting the CAM messages to FVs by updating the communication link to the RSU. The key objectives are listed as follows:

- To model MEC-Aided Platooning to enhance autonomous driving in ITS. A computation offloading method is proffered to handle the latency and resource management challenges in platooning.
- To formulate a multi-objective optimization problem for enabling platoons by minimizing the AoI and maximizing the communication reliability in vehicular networks.
- To design a MARL framework over DDPG algorithms to train triple cascaded critics, and a global and a local critic to facilitate spectrum access to the CVs.
- To accomplish the Cascaded Multi-Agent DDPG algorithm to address the overestimation bias issue and better control the estimation bias.
- To demonstrate with experiments the performance of the proposed framework by indicating three-times better convergence, ensuring the mean Age of Information to be less than 10 ms and the transmission probability of CAM message verification to be almost 99% for diverse platoon criteria.

The remainder of the paper is organized as follows. Section 2 discusses the system model of the proposed framework. The mathematical modeling of the optimization problem is formulated in Section 3. Section 3 describes the proposed MARL algorithm in the MEC-Assisted Platooning system with convergence analysis in Section 4. Numerical experiments and results are demonstrated in Section 4, and Section 5 draws a conclusion to the paper. The primary notations and their explanations are listed in Table 1.

## 2. System Model and Problem Formulation

### 2.1. Vehicle Platooning

**Figure 1:**
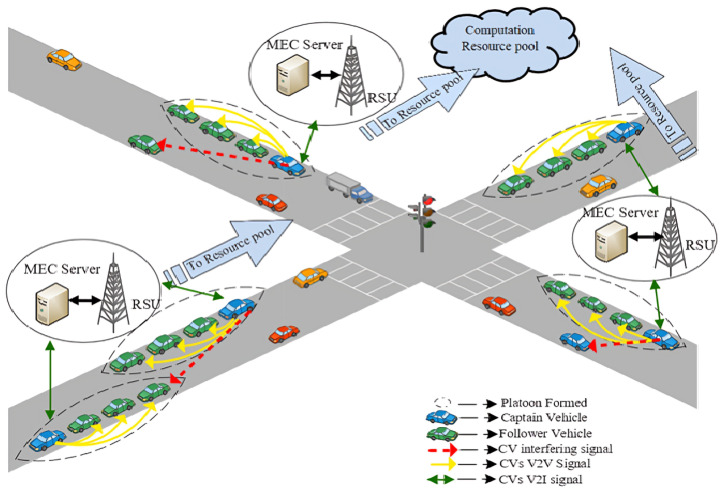
An MEC-Aided multi-platoon architecture.

A cellular V2X Vehicular framework based on Multi-access Edge Computing is considered. The framework comprising multi-platoon architecture and its system model are shown in Figure 1. A wide crossroads scenario with vehicles traveling on a twin-track is presumed. The framework is deployed with RSUs equipped with edge servers positioned in the middle of the crossroad. The group of platoons featured with connected vehicles is denoted as P={1,2,…,P},P∈V. A platoon’s total number of vehicles is given by $V_{i}=\left\{1,2,\ldots ,V_{i}\right\},V_{i}\in V$, and i∈P, which are numerically progressing from CV as one to $V_{i}$. The available time slots are scheduled equally in length Δt, indexing to a positive integer t∈V, and the bandwidth is orthogonally partitioned into *W*-sized subchannels, indexing to b∈B={1,2,…,B}. Essentially, a platooning system has two modes of communication:

- Inter-platoon communication;
- Intra-platoon communication.

In the inter-platoon mode, V2I links are utilized for communicating safety and control information from RSU to all the other platoons. This communication mode is crucially responsible for informing the status of other platoons and crossroads traffic scenarios among all the platoons. However, in intra-platoon mode, V2V links are utilized for communicating the CAM messages between the connected vehicles in each platoon at regular intervals to guarantee familiarity with dynamics and decisions of associated platoon members and platoon captains in particular. The vital purpose of this mode is to achieve the property of string stability in platoons, i.e., the perpetuating distortions are not intensified throughout the string of vehicles. The technical report on 3GPP guidelines [5] states that the broadcasting frequency of CAMs ought to be 100 Hz for driving maneuver coordination, and the CAM message size is denoted as ${ℵ}_{i}$. Orthogonal Frequency Division Multiplexing (OFDM) is leveraged to address the challenges of frequency selective channels. Subsequently, it is assumed that the channel fading is constant within a single subchannel and independent throughout all other subchannels. The channel gain of CV in *b* subchannel within a single coherent time t is modeled based on the occurrence of $\alpha_{i}^{t}$, large-scale fading and $g_{i}^{t}$, small-scale fading as

(1) $$h_{i}^{t}\left[b\right]=\alpha_{i}^{t}g_{i}^{t}\left[b\right]$$

Furthermore, the Boolean factor $\xi_{i,b}^{t}$ that features the allocation of specific subchannel *b* to the *i*th platoon at time slot *t* is desired by $\xi_{i,b}^{t}\in \left\{0,1\right\}$. Indeed, CVs prefer the decision of utilizing the allocated spectrum either for communicating with the RSU or for CAM transmissions to the platoon members, i.e., for inter-platoon or intra-platoon communication, respectively. Subsequently, CVs’ communication mode decision is construed by an additional Boolean factor $\theta_{i}^{t}\in \left\{0,1\right\}$, where 0 symbolizes the decision of the allocated spectrum being utilized for inter-platooning and 1 symbolizes intra-platooning communication.

Based on Claude Shannon’s capacity theorem, the instantaneous rate attained in vehicular communication between CV and the RSU is defined as

(2) $$\begin{array}{cc} \; & C_{i,L}^{t}\left[b\right]=\log_{2}\left(1+\frac{\left(1-\theta_{i}^{t}\right)\xi_{i,b}^{t}p_{i}^{t}\left[b\right]h_{i,L}\left[b\right]}{I_{i}^{t}\left[b\right]+\sigma^{2}}\right),\\ \; & \mathrm{where},\\ \; & I_{i}^{t}\left[b\right]=\sum_{i^{\prime }}\xi_{i^{\prime },b}^{t}p_{i}^{t}\left[b\right]h_{i^{\prime },L}\left[b\right],i\neq i^{\prime } \end{array}$$

where the other platoons’ interference is considered as noise; the CV’s transmitting power level *i* on *b* bandwidth is given as $p_{i}^{t}\left[b\right]$; the CV’s channel gain from *i* to RSU in the bandwidth *b* is given as $h_{i,L}\left[b\right]$; and the noise power and the RSUs locations are defined as $\sigma^{2}$ and L, respectively. The interfering channel from CV’s $i^{\prime }$ performs inter-platoon $\left(\theta_{i}^{t}=0\right)$ or intra-platoon $\left(\theta_{i}^{t}=0\right)$ modes of communication to the RSU, where i∈P and the total interference power is given as $I_{i}^{t}\left[b\right]$.

Consequently, the CVs’ *i* and their Follower Vehicles’ *j* instantaneous rates are defined as

(3) $$\begin{array}{cc} \; & C_{i,L}^{t}\left[b\right]=\log_{2}\left(1+\frac{\theta_{i}^{t}\xi_{i,b}^{t}p_{i}^{t}\left[b\right]h_{i,j}^{t}\left[b\right]}{I_{i}^{\prime ,t}\left[b\right]+\sigma^{2}}\right),\\ \; & \mathrm{where},\\ \; & I_{i}^{\prime ,t}\left[b\right]=\sum_{i^{\prime }}\xi_{i^{\prime },b}^{t}p_{i}^{t}\left[b\right]h_{i^{\prime },j}^{t}\left[b\right],i\neq i^{\prime },j=V_{i} \end{array}$$

The channel gain from a CV *i* to its FV members in the bandwidth *b* is given as $h_{i,j}^{t}\left[b\right]$; the channel interference created from CV $i^{\prime }$s vehicle to the CV *j*’s members, where the CV performs two modes of communication, are given as $h_{i^{\prime },j}^{t}$; finally, the total interference power is given as $I_{i}^{\prime ,t}\left[b\right]$.

As previously stated, the CVs are liable for establishing the timing accuracy while transferring the CAM messages to the RSU. In such scenarios, the Age of Information (AoI) of platoons is aggregated as $AoI_{i}^{t}$ until the scheduling window *t* starts, i.e., the amount of time lapsed from the last effective V2I communication. The $AoI_{i}^{t}$ progresses, following to

(4) $$\begin{array}{c} AoI_{i}^{t+1}=\left\{\begin{array}{cc} \Delta t, & \mathrm{if}\;\left(1-\theta_{i}^{t}\right)\xi_{i,b}^{t}\cdot C_{i,L}^{t}\left[b\right]⩾C_{i,L}^{min}\\ AoI_{i}^{t}+\Delta t, & \mathrm{otherwise} \end{array}\right. \end{array}$$

where the minimum capacity demanded by any reliable vehicular communication is represented as $C_{i,L}^{min}$, and whenever the RSU and CV successfully communicate the data, the $AoI_{i}^{t}$ restarts from Δt.

### 2.2. MEC-Aided Platooning

An MEC-Aided platooning framework with its communication paradigm is shown in Figure 1. As discussed earlier, the E edge nodes are installed along the crossroads and are denoted as $E=\{e_{1},e_{2},e_{3},\ldots ,e_{n}\}$. The effective process of content caching at the edges of the vehicular network is capable of relieving the load on the backhaul links and hastening the transmission. Hitherto, the concept of caching space and computational resources is deployed along the *n*th edge node and is denoted as $es_{n}$ and $er_{n}$, respectively. The individual connected vehicles V have *i* computing tasks that are being offloaded to edge nodes. The computing tasks are represented as ct and are given by $CT=\{ct_{1},ct_{2},\ldots ,ct_{i}\}$. These computational tasks demand caching space and computational resource needs denoted as $ct_{sn}$ and $cr_{rn}$, respectively. The RSU installed along the crossroads is given by $RSU=\{RSU_{1},RSU_{2},\ldots ,RSU_{n}\}$. The physical resources of the edge nodes are considered to be resource blocks with equal processing power ${\mathrm{Pp}}_{n}$. The time of execution is entirely defined by the block’s capacity $C_{n}$, the task’s required amount of resource block $U_{n}$, and the required data rate.

#### Latency Model

Latency becomes involved as the edge nodes begin the execution of task computation, comprising distinct transmission times such as the following:

- The time for a transmission from the initial vehicle to the intended vehicle;
- The time for task offloading from the intended vehicle to the destination edge node;
- The time for task execution on the edge node destination;
- The time for responding to the computational estimations from edge nodes to the initiated vehicle.

On the assumed crossroads, each of the vehicles traverse various edge nodes. Considering that Poisson distribution characterizes the vehicle distribution, the reception of task offloading at the MEC server also follows a Poisson process. In order to guarantee the presence of the vehicle at the destination edge node’s $e_{n}$ coverage at the specific instant *x*, another Boolean factor $\phi_{i}^{n}\left(x\right)$ is used for discovering the location of the vehicle $V_{i}^{\prime }$ and is determined by

(5) $$\begin{array}{c} \phi_{i}^{n}\left(x\right)=\left\{\begin{array}{cc} 0, & V_{i}\;\mathrm{is}\;\mathrm{in}\;\mathrm{the}\;\mathrm{coverage}\;\mathrm{of}\;e_{n}\\ 1, & \mathrm{otherwise} \end{array}\right. \end{array}$$

The transmission of tasks originates from the initiated vehicle to the intended vehicle that is in the vicinity of the edge node through the multi-hop V2V technique. The primary cause of latency is contributed by computation task transmission time ${trans}_{i}\left(x\right)$ and is obtained by

(6) $$\begin{array}{c} {trans}_{i}\left(x\right)=\sum_{n=1}^{N}\sum_{i^{\prime }=1}^{I}\phi_{i}^{n}\cdot \left(1-\phi_{i^{\prime }}^{n}\right)\cdot Y_{i}^{i^{\prime }}\cdot \frac{d_{i}}{C_{i,j}^{t}\left[b\right]}\cdot \left(x_{i^{\prime }}+1\right) \end{array}$$

in which $d_{i}$ is the task offloaded data size and $C_{i,j}^{t}\left[b\right]$ is the rate at which data are transmitted in the network by applying V2V techniques; also, based on the literature, it is assumed to be a constant since V2X users’ data transmission occurs in fixed power. $x_{i’}$ is the vehicle number through which the task is traversed from $v_{i}$ to $v_{i’}$. On the other hand, $Y_{i}^{i’}$ represents the Boolean factor to determine if $v_{i’}$ is the vehicle destination and is determined as

(7) $$\begin{array}{c} Y_{i}^{i^{\prime }}\left(x\right)=\left\{\begin{array}{cc} 0, & V_{i^{\prime }}\;\mathrm{is}\;\mathrm{the}\;\mathrm{destination}\;\\ 1, & \mathrm{otherwise} \end{array}\right. \end{array}$$

The secondary cause of latency is contributed by *i*th task offloading time $off_{i}\left(x\right)$ at the data rate $C_{i,L}^{t}\left[b\right]$—the rate between the vehicle and the edge nodes based on V2I technique—and is obtained as

(8) $$\begin{array}{c} {off}_{i}\left(x\right)=\sum_{e=1}^{N}\phi_{i^{\prime }}^{n}\left(x\right)\cdot \frac{d_{i}}{C_{i,L}^{t}\left[b\right]} \end{array}$$

The tertiary cause of latency is due to the *i*th task execution time executed on the destination edge node. Let us consider the edge node’s resources as physical resource units and the task execution time as decided by the task’s data size. The capacity of the edge node is represented by $C_{n}$, the number of units needed to execute the task is denoted by $U_{n}$, and the processing power required by all the resources for executing the task is constant and given by ${\mathrm{Pp}}_{n}$. The time for task execution is determined by

(9) $$\begin{array}{c} {exec}_{i}\left(x\right)=\sum_{e=1}^{N}\left(1-Y_{i}^{i^{\prime }}\left(x\right)\right)\cdot \frac{C_{n}}{U_{n}\cdot {\mathrm{Pp}}_{n}} \end{array}$$

Consequently, the final cause of latency is the responding time of offloading estimations returned to the initiated vehicles from the edge nodes leveraging V2I technology and is determined as

(10) $$\begin{array}{c} {resp}_{i}\left(x\right)=\frac{d_{i^{\prime }}}{C_{i,L}^{t}\left[b\right]} \end{array}$$

The overall latency $l_{i}\left(x\right)$ required for the $ct_{i}$ task execution is determined as

(11) $$\begin{array}{c} l_{i}\left(x\right)={trans}_{i}\left(x\right)+{off}_{i}\left(x\right)+{exec}_{i}\left(x\right)+{resp}_{i}\left(x\right) \end{array}$$

Subsequently, the total latency *L* for the overall execution of task computations is estimated as

(12) $$\begin{array}{c} L=\sum_{i=1}^{I}l_{i}\left(x\right) \end{array}$$

### 2.3. Problem Formulation

In this section, the Multi-Objective Optimization Problem (MOOP) is realized by concurrently minimizing the AoI, maximizing the CAM message Delivery Rate Ratio (DRR) probability, and minimizing the latency. The MOOP is precisely defined as

minAoI,maxDRR,minL

subject to

(13) $$\begin{array}{ccc} C_{1}: & C_{i,L}^{t}\left[b\right]⩾C_{i,L}^{min} & \forall i\in P,\forall b\in B\\ C_{2}: & \xi_{i,b}^{t},\theta_{i}^{t}\in \left\{0,1\right\} & \forall i\in P,\forall b\in B\\ C_{3}: & \sum_{b\in B}\xi_{i,b}^{t}⩽1 & \forall i\in P,\forall t\in V\\ C_{4}: & V_{i}\in er_{n} & \forall i\in \{1,2,\ldots ,I\},n\in \{1,2,\ldots ,N\}\\ C_{5}: & \sum_{i=1}^{I}ct_{i}⩽\sum_{n=1}^{N}es_{n}; & \forall i\in \{1,2,\ldots ,I\},n\in \{1,2,\ldots ,N\}\\ \; & \sum_{i=1}^{I}cr_{i}⩽\sum_{n=1}^{N}er_{n} & \end{array}$$

The objective functions are estimated to minimize the AoI and maximize the delivery ratio rate probability of CAM messages for every T seconds amid the platoon vehicles while minimizing the latency as a consequence of computing task offloading. The data rate between the CV and RSU should not be significantly less than the minimum required capacity of the CV taken into consideration in c1. In the estimated optimization function, the mode and subchannel selection criteria are implied as Boolean factors in $C_{2}$. According to constraint $C_{3}$, one particular subchannel can be utilized by every platoon over a given period of T. The actual amount of computational resources and caching spaces needed for the task execution are beyond the resources delivered by the edge nodes and are ensured by constraint $C_{5}$.

It becomes challenging to address the mixed-integer nonlinear programming problem that was presented in (13) due to the following factors. Binary integers are used for denoting the decision of utilizing the allocated resources, representing the chosen mode of communication, discovering the location of the vehicle in the vicinity area, and indicating the vehicle destination in the V2V communication. These factors can lead to a combinatorial decision issue. Furthermore, the defined constraints are non-convex problems offering local numerical optimum solutions. The centralized approach employed in C-V2X-based communication in the literature [30,31] involves high computational complexity restricting the scalability of vehicular networks.

However, the objective function becomes a challenging, non-convex, and NP-hard optimization problem. In order to combat the complexity of the suggested optimization techniques, the cutting-edge Multi-Agent Deep Deterministic Policy Gradient approach is analyzed.

## 3. Resource Allocation Based on a Multi-Agent Reinforcement Learning Algorithm

In this section, the vehicular environment associated with the agent in the multi-agent scenario comprising state, action, and rewards is attempted for demonstration. Subsequently, a novel CMADDPG algorithm for addressing the estimation bias issue in Q-Learning is proposed with its appropriate equations.

### 3.1. Multi-Agent Environment Modeling

The optimization problem for Multi-Agent Reinforcement Learning with *P* agents from the platoons can be demonstrated as

(14) $$\begin{array}{c} \max_{\pi_{i}}Z_{i}\left(\pi_{i}\right),\;i\in P,\pi_{i}\in \Pi_{i}, \end{array}$$

$$\begin{array}{c} Z_{i}\left(\pi_{i}\right)=E\left[\sum_{t=0}^{\infty }\gamma^{t}R_{i}^{t+1}|S_{i}^{0}\right],\pi_{i} \end{array}$$

in which $Z_{i}\left(\pi_{i}\right)$ is agent *i*’s policy and $\Pi_{i}$ is agent *i*’s possible array of policies. Each CV employed as an agent communicates with the environment. In compliance with the policy, the agent decides on actions to maximize the expected rewards. At every time slot *t*, the current state $s^{t}$ is observed by CV and decides the action $a^{t}$ corresponding to its policy. As the environment evolves to the next state $s^{t+1}$, the CV is rewarded for the chosen action. The proposed MADDPG system is modeled as follows:

State space S;
Action space A;
Immediate reward R.

#### 3.1.1. State Space

At a specific time slot t, the CV acting as an agent observes the current state S and the observation comprises multiple factors as follows:

- The present channel gain knowledge between the CV and RSU indicated as $h_{i,L}^{t}\left[b\right]$;
- The channel gain knowledge between the CV and its FV indicated as $h_{i,j}^{t}\left[b\right]$;
- The prior knowledge of interference to the CV from another platoon member indicated as $I_{i}^{t-1}\left[b\right]$;
- The AoI of CV indicated as $AoI_{i}^{t}$;
- The dissemination of residual CAM messages in the specified time limit T⩽100 ms indicated as ${ℵ}_{i}^{r}$;
- The residual estimated time indicated as $T_{i}^{r}$.

Consequently, the CV *i*’s agent state space, where $s_{i}^{t}\in S$, is given by

$$\begin{array}{c} s_{i}^{t}=\left\{h_{i,L}^{t}\left[b\right],h_{i,j}^{t}\left[b\right],I_{i}^{t-1}\left[b\right],AoI_{i}^{t},{ℵ}_{i}^{r},T_{i}^{r}\right\}\;i\in P \end{array}$$

#### 3.1.2. Action Space

The ideal action space A for the proposed environment must include every subchannel allocation decision. Indeed, the action space $a_{i}^{t}$ is featured as follows:

- The subchannel decision made by the CV indicated as $\xi_{i}^{t}$;
- The mode of communication decision made by the CV indicated as $\theta_{i}^{t}$.

Therefore, the CV’s action space, where i∈P and $a_{i}^{t}\in A$, can be given as

$$\begin{array}{c} a_{i}^{t}=\left\{\xi_{i}^{t},\theta_{i}^{t}\right\} \end{array}$$

#### 3.1.3. Immediate Reward

The most insightful factor among the Reinforcement Learning algorithms is the potential it holds for defining the reward function R for actuating the learning process. The immediate reward can be defined as R:S×A, and r(s,a) is the real value immediate reward as the current state *s* is exposed to the action *a*. In the proposed novel CMADDPG algorithm, two reward signals are defined: global reward and immediate local reward. The global rewards received by the agent are responsible for estimating the agent’s level of cooperation, while the local rewards are responsible for evaluating the effectiveness of every agent individually.

### 3.2. Conventional Multi-Agent Deep Deterministic Policy Gradient

**Figure 2:**
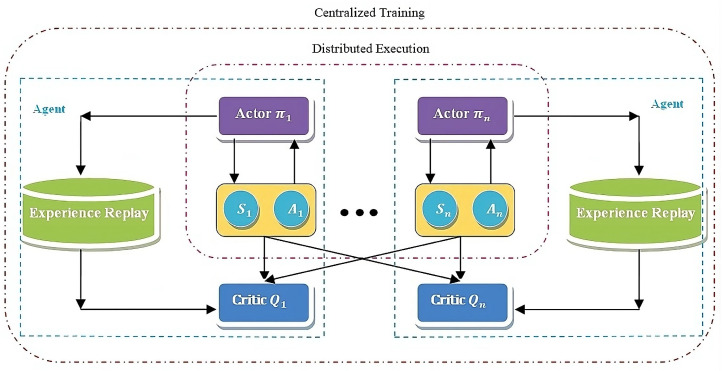
The MADDPG framework.

The key concepts of MADDPG is illustrated in Figure 2, Based on the MADDPG principle, two networks are present in every agent: the actor network π and the critic network *Q*. The actor network is responsible for computing the executed actions depending on the states obtained by the agent, whereas the critic network is responsible for estimating the action computed by the actor network in an effort to enhance the actor network’s efficiency. To eliminate the redundancies in the training data and improve the training stability, a particular portion of experiences obtained from training are stored in a buffer called the Experience Replay buffer, denoted by D, from which the critic network randomly selects while updating the network. During the training phase, the actor network solely learns from its own observations; concurrently, the critic network learns from data, which include the actions and observations of additional agents. During the execution phase, the agent mandates the participation of the actor network alone, hence signifying the decentralized execution.

**Figure 3:**
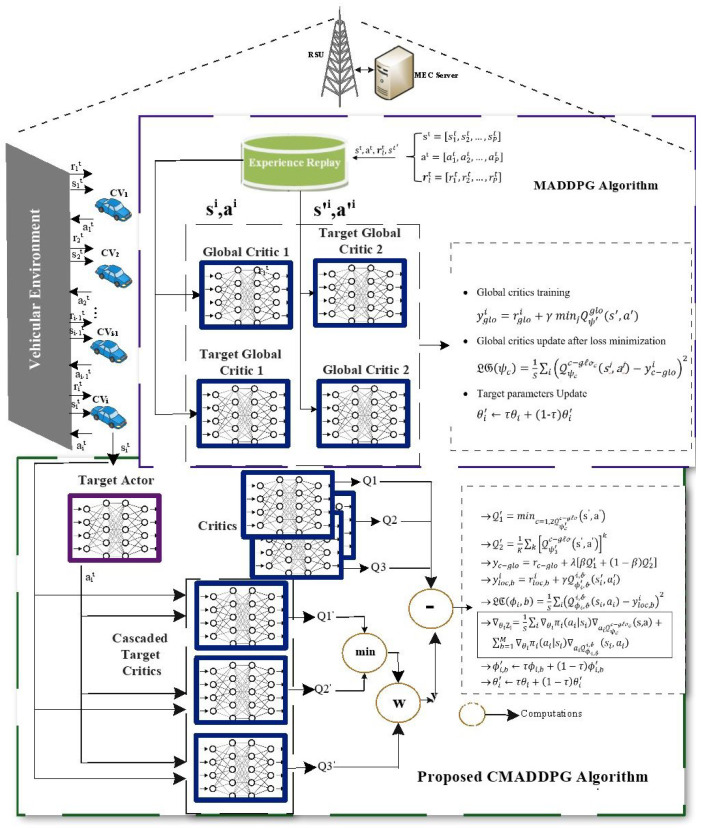
The CMADDPG architecture.

Let us consider $\Theta_{\pi }=\left(\omega_{\pi }^{\left(1\right)}\ldots \omega_{\pi }^{\left(L_{\pi }\right)}\right)$ and $\Phi_{Q}=\left(\omega_{Q}^{\left(1\right)}\ldots \omega_{Q}^{\left(L_{Q}\right)}\right)$ as the agent’s space parameters of the actor network and critic network. Let the global critic’s space parameter be $\Psi_{glo}=\left(\omega_{glo}^{\left(1\right)}\ldots \omega_{glo}^{\left(L_{glo}\right)}\right)$, in which the quantity of hidden layers in the agent’s actor, critic, and global critic is given as $L_{\pi },L_{Q},L_{glo}$, respectively, with ω being the weights of the neural network. The agent’s policies for the Vehicular platooning scenario comprising *P* platoons as agents are given as $\pi =\pi_{1},\ldots ,\pi_{P}$. The Q-functions, global critic’s Q-function, and the agent’s policies are given as $Q_{\phi_{i}}^{i},Q_{\psi }^{glo},\pi_{i}$, and characterized as $\phi_{i},\psi ,\theta_{i}$, respectively, in which $\phi_{i}\in \Phi_{Q},\psi \in \Psi_{glo}$, and $\theta_{i}\in \Theta_{\pi }$. The MADDPG for the platoon *i* is defined as

(15) $$\begin{array}{c} \nabla_{\theta_{i}}Z_{i}=E\left[\nabla_{\theta_{i}}\pi_{i}\left(a_{i}|s_{i}\right)\nabla_{a_{i}}Q_{i}^{\pi }\left(s,a\right){|}_{a_{i}=\pi_{i}\left(s_{i}\right)}\right], \end{array}$$

where the absolute state and the absolute action spaces are given by $s=\left(s_{1},s_{2}\ldots ,s_{P}\right)$ and $a=\left(a_{1},a_{2},\ldots ,a_{P}\right)$, respectively, and the centralized action-value function is given by $Q_{i}^{\pi }\left(s,a\right)$. The Q-value for platoon *i* is determined by feeding the states and actions of the agent to the centralized function. Formulated from the architecture illustrated in Figure 3, the general Multi-Agent Deep Deterministic Policy Gradient for an individual agent with twin critics is given as

(16) $$\begin{array}{c} \nabla_{\theta_{i}}Z_{i}=\underset{Critic_{Global}}{\underset{︸}{E_{s,a∼D}\left[\nabla_{\theta_{i}}\pi_{i}\left(a_{i}|s_{i}\right)\nabla_{a_{i}}Q_{\psi }^{glo}\left(s,a\right)\right]}}+\underset{Critic_{Local}}{\underset{︸}{E_{s,a∼D}\left[\nabla_{\theta_{i}}\pi_{i}\left(a_{i}|s_{i}\right)\nabla_{a_{i}}Q_{\phi_{i}}^{i}\left(s_{i},a_{i}\right)\right]}} \end{array}$$

in which the well-fitting action picked by the action *i* supervening its policy $\pi_{i}$ is defined as $a_{i}^{t}=\pi_{i}\left(s_{i}^{t}\right)$. The term $Critic_{Global}$ in Equation (16) evaluates the team reward based on i’s state and action; however, the term $Critic_{Local}$ evaluates the performance of each agent by exclusively considering the local state and action of an individual agent. The term $Critic_{Global}$ is updated as

(17) $$\begin{array}{c} \mathrm{LG}\left(\psi \right)=E_{s,a,r,s^{\prime }}\left[{\left(Q_{\psi }^{glo}\left(s,a\right)-y_{glo}\right)}^{2}\right] \end{array}$$

and the target value $y_{glo}$ is interpreted as

(18) $$\begin{array}{c} y_{glo}=r_{glo}+\gamma Q_{\psi^{\prime }}^{glo}\left(s^{\prime },a^{\prime }\right){|}_{a_{i}^{\prime }=\pi_{i}^{\prime }\left(s_{i}^{\prime }\right)} \end{array}$$

in which the target policies $\pi^{\prime }=\left\{\pi_{1}^{\prime },\pi_{2}^{\prime },\ldots ,\pi_{P}^{\prime }\right\}$ are specified as $\theta^{\prime }=\left\{\theta_{1}^{\prime },\theta_{2}^{\prime },\ldots ,\theta_{P}^{\prime }\right\}$. Correspondingly, the term $Critic_{Local}$ is updated as

(19) $$\begin{array}{c} {\mathrm{LC}}^{i}\left(\phi_{i}\right)=E_{s_{i},a_{i},r_{i},s_{i}^{\prime }}\left[{\left(Q_{\phi_{i}}^{i}\left(s_{i},a_{i}\right)-y_{loc}^{i}\right)}^{2}\right] \end{array}$$

and $y_{loc}^{i}$ is given as

(20) $$\begin{array}{c} y_{loc}^{i}=r_{loc}^{i}+\gamma Q_{\phi_{i}^{\prime }}^{i}\left(s_{i}^{\prime },a_{i}^{\prime }\right){|}_{a_{i}^{\prime }=\pi_{i}^{\prime }\left(s_{i}^{\prime }\right)} \end{array}$$

### 3.3. The Bias Estimation Phenomenon

Even though DQN is capable of handling several competitive problems, there remain unsolved issues; hence, to enhance the performance, numerous solutions have been suggested. However, the suggested techniques can support discrete actions only and are incapable of handling real-world continuous action scenarios. In relevance, various policy gradient techniques are featured, and one significant technique is the DDPG, which utilizes a deterministic policy in the actor–critic-based method. The action-value function or Q-function manages to estimate the policy and facilitates learning the optimal best policy to achieve the highest value. When the transition kernel is uncertain, Q-functions are iteratively approximated based on the Bellman equation. Consequently, such function approximations are subject to estimation variance as well as estimation bias, owing to generalization and value iteration, respectively. In addition, Twin Delayed Deep Deterministic Policy Gradient (TD3), an immense expansion of DDPG, initiates three strategies—namely, Clipped Double-Q Learning, “Delayed” Policy Updates, and Target Policy Smoothing. In this algorithm, either of the critics who are underestimated is chosen instead of an overestimated critic to mitigate the overestimation. In order to reduce overfitting, noise is included in the target policy in the smoothing procedure, which, regretfully, leads to erroneous function approximation. Eventually, the erroneous approximation and the action of minimization over the twin critics induce underestimation bias.

### 3.4. The Proposed Cascaded Multi-Agent Deep Deterministic Policy Gradient

Despite the fact that the abovementioned framework produces fair outcomes, the overestimation bias problem persists as a consequence of function approximation and the twin critic’s minimization issues. The policy gradient algorithms generate overestimation bias, whereas TD3 algorithms generate underestimation bias. Subsequently, a novel Cascaded Multi-Agent Deep Deterministic Policy Gradient (CMADDPG) is proposed that utilizes the two contrasting biases to produce further precision in the estimation. The CMADDPG architecture depicted in Figure 3 is described in Algorithm 1. While a single-critic DDPG delivers overestimation and twin critics deliver underestimation, tri critics are employed in a cascaded mode to accomplish reliable predictions. Therefore, the architecture of the Cascaded Multi-Agent Deep Deterministic Policy Gradient (CMADDPG) algorithm was designed on the pinnacle of the DDPG and TD3 framework, as shown in Figure 3. In the proposed CMADDPG algorithm, the target value is updated by determining the minimum Q-value between the two critics followed by a Weighting operation over the obtained minimum value, and the Q-value of the third critic.

Consequently, the existing $Critic_{Global}$ is substituted by Cascaded Tri critics c={1,2,3}, and the policy gradient outcome is given by

(21) $$\begin{array}{c} \nabla_{\theta_{i}}Z_{i}=\underset{Critic_{Cascaded\;Global}}{\underset{︸}{E_{s,a∼D}\left[\nabla_{\theta_{i}}\pi_{i}\left(a_{i}|s_{i}\right)\nabla_{a_{i}}Q_{\psi_{c}}^{{c-glo}_{c}}\left(s,a\right)\right]}}+\underset{Critic_{Local}}{\underset{︸}{E_{s_{i},a_{i}∼D}\left[\nabla_{\theta_{i}}\pi_{i}\left(a_{i}|s_{i}\right)\nabla_{a_{i}}Q_{\phi_{i}}^{i}\left(s_{i},a_{i}\right)\right]}} \end{array}$$

where (s,a) are the total state and action spaces. $Q_{\psi_{c}}^{{c-glo}_{c}}$ and $Q_{\phi_{i}}^{i}$ are the centralized action-value functions that estimates the Q-value for platoon *i* using the agents’ states and actions as input. The primary term in (21) corresponds to the cascaded global critic, which assesses the team reward based on the states and actions of the agents. The secondary term in (21) refers to the local critic of each agent, which, in contrast to the cascaded global critic, solely considers the local state and activity of each agent to rate the performance of each agent individually.

The $Critic_{Cascaded\;Global}$ is updated as

(22) $$\begin{array}{c} \mathrm{LG}\left(\psi_{c}\right)=E_{s,a,r,s^{\prime }}\left[{\left(Q_{\psi_{c}}^{{c-glo}_{c}}\left(s,a\right)-y_{c-glo}\right)}^{2}\right] \end{array}$$

and the target value $y_{c-glo}$ is given as

(23) $$\begin{array}{c} y_{c-glo}=r_{c-glo}+\gamma \left\{\beta \min_{c=1,2}Q_{{\psi^{\prime }}_{c}}^{{c-glo}_{c}}\left(s^{\prime },a^{\prime }\right)+\left(1-\beta \right)Q_{{\psi^{\prime }}_{3}}^{{c-glo}_{3}}\left(s^{\prime },a^{\prime }\right)\right\}{|}_{a_{i}^{\prime }=\pi_{i}^{\prime }\left(s_{i}^{\prime }\right)} \end{array}$$

in which the twin critics’ weight is denoted by β∈{0,1} and the update of local critics is analogous to the global critics. In (23), $\pi_{i}^{\prime }$ refers to the target policies.

In the course of implementation, a $Q_{1}$ optimized single actor is utilized, and the identical target values are fixed for $Q_{1},Q_{2}$, and $Q_{3}$. In every update, $Q_{c}$ where c=1,2 is considered as an unbiased estimation. Similarly, $Q_{3}$ is considered to be overestimated with the DDPG bias by assuming the fact that DDPG encourages overestimation in all the scenarios. The expected bias estimation of CMADDPG with the impact of the weighting factor β can be written as

(24) $$\begin{array}{c} E\left[EB^{CMADDPG}\right]=-\frac{1}{3}\beta \gamma \epsilon +\left(1-\beta \right)\lambda \end{array}$$

Primarily focusing on the mentioned considerations, it is evident that the proposed bias estimation is beyond TD3 but lower than DDPG, demonstrating unbiased estimation through fair hypotheses and favorable decisions on weight.

### 3.5. Formulation of Rewards

The designing of well-balanced rewards becomes essential for successfully addressing the optimization challenges (13). In the considered scenario, each CV agent attempts to utilize the accessible subchannel resource for two distinct purposes: (i) To minimize the AoI by updating the communication to the RSU; (ii) To reliably deliver the CAM messages to the FVs. The local reward for each platoon *i* is given as

(25) $$\begin{array}{c} r_{loc}^{i}=\underset{intra-platoon}{\underset{︸}{-\{w_{1}{ℵ}_{i}^{r}{ℵ}_{i}\}}}\underset{inter-platoon}{\underset{︸}{-w_{2}AoI_{i}^{t}+w_{3}G\left(C_{i,L}^{t}-C_{i,L}^{min}\right)}} \end{array}$$

In Equation (25), weighting parameters represented as $w_{1}$–$w_{4}$ are applied to optimize the rewards. The optimization of rewards is formulated to reflect the constraints of the objectives. The initial component corresponds to the rewards obtained for communicating in intra-platoon mode, and the later component corresponds to the rewards for the inter-platoon communication mode. Similarly, the global reward can be formulated as

(26) $$\begin{array}{c} r_{glo}^{i}=-\frac{1}{P}\sum_{i\in P}\sum_{b\in B}\log_{10}\left\{I_{i}^{t}\left[b\right]\right\} \end{array}$$

The inclination of platoons to discover the subchannels that elicit minimized interference toward other platoons motivates the formulation of global rewards focusing on approximating the mean interference. The revenue *R* is desired to be a positive integer indicated by a piecewise constant function G(x) in Equation (25).

$$\begin{array}{c} G\left(x\right)=\left\{\begin{array}{cc} R, & x\geq 0\\ 0, & x<0 \end{array}\right. \end{array}$$

**Algorithm 1** CMADDPG
**Input:** Observation state S**Output:** Action AStart generating platoons from environment simulatorInitialize main global critics $Q_{\psi_{1}}^{{c-glo}_{1}},Q_{\psi_{2}}^{{c-glo}_{2}}$ and $Q_{\psi_{3}}^{{c-glo}_{3}}$Initialize target global critics $Q_{\psi_{1}^{\prime }}^{{c-glo}_{1}},Q_{\psi_{2}^{\prime }}^{{c-glo}_{2}}$ and $Q_{\psi_{3}^{\prime }}^{{c-glo}_{3}}$Initialize policy networks and critic networks defined by task of every agentInitialize Experience Replay Buffer D with *K* previous networks ${\left[Q_{\psi_{3}^{\prime }}\right]}^{1},\ldots ,{\left[Q_{\psi_{3}^{\prime }}\right]}^{K}$**for** *each episode* **do**    Receive initial observation state $s_{1}$    Update location and channel gains of each platoon    Reset the CAM message size *ℵ* and time limit *T* determined as 100 ms    **for** *each time slot t* **do**        **for** *each agent b* **do**           Observe $s_{b}^{t}$ and choose action $a_{b}^{t}=\pi_{\theta_{b}}\left(s_{b}^{t}\right)+\epsilon$ based on the current policy           and exploration noise ϵ∈N(0,σ)        **end for**        $s^{t}=\left[s_{1}^{t},s_{2}^{t},\ldots ,s_{P}^{t}\right]$ and $a^{t}=\left[a_{1}^{t},a_{2}^{t},\ldots ,a_{P}^{t}\right]$        Receive global and local rewards, $r_{glo}^{t}$ and $r_{loc}^{t}$        Store transition $\left(s^{t},a^{t},r_{glo}^{t},r_{loc}^{t},s^{t+1}\right)$ in Experience Replay Buffer D    **end for**    Sample random mini-batch of S transitions $\left(s^{i},a^{i},r_{glo}^{i},r_{loc}^{i},s^{\prime i}\right)$ from D    Set $a_{b}^{t}=\pi_{\theta_{b}}\left(s_{b}^{t}\right)+\epsilon ,\epsilon ∼$ clip $\left(N\left(0,\tilde{\sigma }\right),-c,c\right)$    Set $Q_{1}^{\prime }=min_{c=1,2}Q_{\psi_{c}^{\prime }}^{c-glo}\left(s^{\prime },a^{\prime }\right)$    Set $Q_{2}^{\prime }=\frac{1}{K}\sum_{k}{\left[Q_{\psi_{3}^{\prime }}^{c-glo}\left(s^{\prime },a^{\prime }\right)\right]}^{k}$    Set $y_{c-glo}=r_{c-glo}+\lambda \left[\beta Q_{1}^{\prime }+\left(1-\beta \right)Q_{2}^{\prime }\right]$    Update global critics by minimizing the loss: $\mathrm{LG}\left(\psi_{c}\right)=\frac{1}{S}\sum_{i}{\left(Q_{\psi_{c}}^{{c-glo}_{c}}\left(s^{i},a^{i}\right)-y_{c-glo}^{i}\right)}^{2}$    **if** *episode mod d* **then**        Train local actor network and critic network        **for** *each agent j* **do**           **for** *each task b* **do**               Set $y_{loc,b}^{i}=r_{loc,b}^{i}+\gamma Q_{\phi_{i}^{\prime },b}^{i,b}\left(s_{i}^{\prime },a_{i}^{\prime }\right)$               Update local critics by minimizing the loss: $\mathrm{LC}\left(\phi_{i},b\right)=\frac{1}{S}\sum_{i}{\left(Q_{\phi_{i},b}^{i,b}\left(s_{i},a_{i}\right)-y_{loc,b}^{i}\right)}^{2}$           **end for**           Update local actor network using deterministic policy gradient:           $\nabla_{\theta_{i}}Z_{i}=\frac{1}{S}\sum_{i}\nabla_{\theta_{i}}\pi_{i}\left(a_{i}|s_{i}\right)\nabla_{a_{i}}Q_{\psi_{c}}^{{c-glo}_{c}}\left(s,a\right)+\sum_{b=1}^{M}\nabla_{\theta_{i}}\pi_{i}\left(a_{i}|s_{i}\right)\nabla_{a_{i}}Q_{\phi_{i,b}}^{i,b}\left(s_{i},a_{i}\right)$                    Update target network parameters:                     **for** *each task b* **do**                        $\phi_{i,b}^{\prime }\leftarrow \tau \phi_{i,b}+\left(1-\tau \right)\phi_{i,b}^{\prime }$            **end for**           $\theta_{i}^{\prime }\leftarrow \tau \theta_{i}+\left(1-\tau \right)\theta_{i}^{\prime }$           Update D by broadcasting the earliest and populating the current target critic network        **end for**    **end if**
**end for**



### 3.6. Computational Complexity

The efficiency of the algorithm critically depends on its computational complexity. Consequently, the complexity of the computations of the proposed MADDPG algorithms is evaluated; thereby, the algorithms are compared with the benchmark RL algorithms adapted widely in the literature. In accordance with the existing studies, the two essential elements that serve for these evaluations are (i) the ratio of trainable variables and (ii) the average number of neural networks involved in the algorithm.

#### 3.6.1. The Ratio of Trainable Variables

Each agent’s state and action are incorporated into the Q-Learning input. In contrast, in MADDPG, every agent has a unique state and action. Let $\hat{s}$ and $\hat{a}$ be the state and action of each agent and the ratio of trainable variables be in the order of $O(v^{2}(\hat{s}$ + $\hat{a}))$, where v refers to the number of vehicles or agents. The proposed CMADDPG algorithm comprises both global and local critics, and the algorithm utilizes a globally centralized Q-function, in which the range of variables grows in the order $O(v(\hat{s}$ + $\hat{a}))$. Subsequently, the variable range for the local critic remains in the order $O(\hat{s}$ + $\hat{a})$ for the reason that the input of the local critic will be the state and action of their corresponding vehicle agent.

#### 3.6.2. The Average Amount of Neural Networks

The magnitude of the neural network utilized in the training phase of traditional MADDPG is double that of $\left(v\left({\underline{1}}_{Q}+{\underline{1}}_{A}\right)\right)$. The utilization of the target network doubles the magnitude, and the actor and critic of every agent are given by ${\underline{1}}_{A}$ and ${\underline{1}}_{Q}$, respectively. The proposed CMADDPG involves triple global critics and, hence, the magnitude of the neural network is given by $2\times (v\left({\underline{1}}_{Q_{l}}+{\underline{1}}_{A_{l}}\right)+3{\underline{1}}_{Q_{g}})$, where the local critics are represented as ${\underline{1}}_{Q_{l}}$ and ${\underline{1}}_{A_{l}}$ and global critics are represented as ${\underline{1}}_{Q_{g}}$

## 4. Experimental Results and Analysis

In this section, the performance of the proposed CMADDPG-based spectrum resource allocation algorithm in Platooning Vehicular networks is evaluated and results are closely analyzed. On a highway, a wide crossroads scenario with vehicles traveling on a twin track is presumed based on [9] that delivers the vehicle velocity, the vehicle’s travel direction together with V2I and V2V channel modeling, path-loss modeling, and shadowing. Small-scale fading is modified while large-scale fading is kept constant for each episode. Moreover, the global reward $r_{glo}^{i}$ in (26) is normalized for accuracy to the same range of local reward. Similarly, the transmit power of each vehicle in the platoon and the Base station determines the speed of offloading between two vehicles, and between the edge nodes and the platoon vehicles, respectively. The simulation and neural network parameters are tabulated in Table 2. Consequently, the effectiveness of the proposed algorithm is determined by employing three benchmark algorithms based on the literature.

- Benchmark 1—MADDPG: In the MADDPG algorithm, the set of agents concurrently learns together to maximize the global and local reward in a cooperative environment. Every platoon in the architecture is equipped locally with a platoon dataset-trained actor and critic network. The RSU comprises global critics that are responsible for regularly criticizing the competence of action preferred by the platoons and also ensures the platoon’s coordination.
- Benchmark 2—Fully Decentralized MADDPG: In the Fully Decentralized MADDPG algorithm, the platoons determine the action depending on the information obtained as a completely decentralized decision rather than counting the global critics.
- Benchmark 3—Fully Centralized DDPG: In the fully centralized DDPG algorithm, it is the responsibility of the RSU to gather all the platoon’s information in order to make decisions about the action to be performed in a centralized manner.

### 4.1. Network Architecture

The network architecture of the proposed CMADDPG algorithm utilizes a fully connected, double-layered neural network with 300 and 400 hidden neurons along with Rectified Linear Units (ReLU) in between every layer. A tanh unit in the last is continued by the actor’s output. The state and action are provided to the initial layers of the critic. A mini-batch consisting of 100 uniformly sampled transitions is selected and updated at a learning rate of $10^{-3}$ by utilizing the Adam optimizer, which in turn is expected to reduce the algorithmic loss. The delay parameter for updating the policy of actor and target critics is assigned to be d=2 iterations with τ=0.005.

The off-policy exploration is leveraged by adding Gaussian noise N(0,0.1) to the actions determined by the target actors in order to trade-off exploitation and exploration; later, the noise is clipped to N(−0.5,0.5) to achieve target policy smoothing. However, exploring tasks are carried out rather than training for experience acquisition. The symmetry between the under and overestimation biases is attained by assigning the weight factor β to 0.95. Similarly, the approximation errors are addressed by calculating the median values of target action *K* for each update, and minimal variation in the K value enhances the stability of continuous actions.

### 4.2. Simulation Results

#### 4.2.1. Performance Comparison of Reward Function Convergence

**Figure 4:**
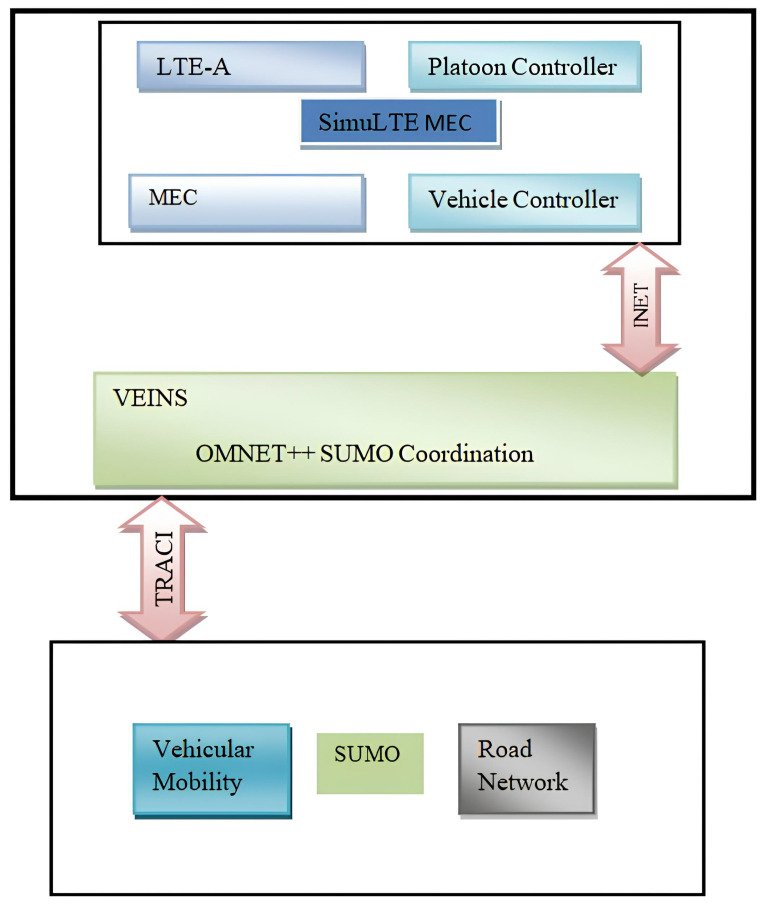
Simulation framework.

**Figure 5:**
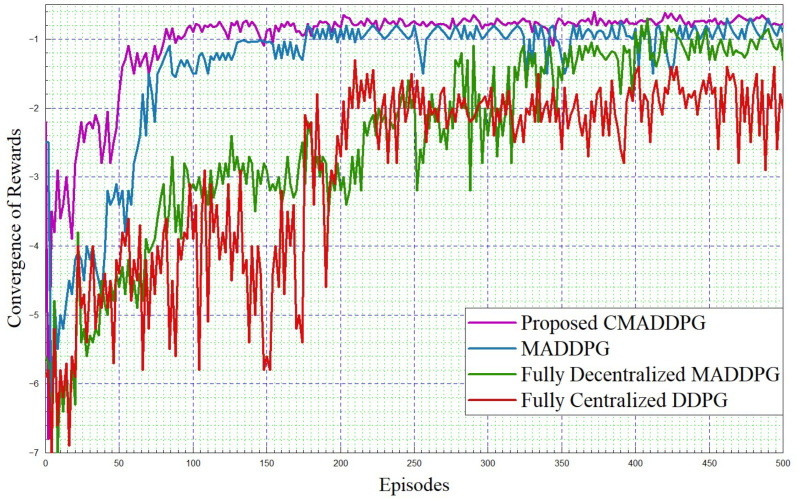
Convergence of rewards. P=7,V=5, intra-platoon spacing =5 m.

**Figure 6:**
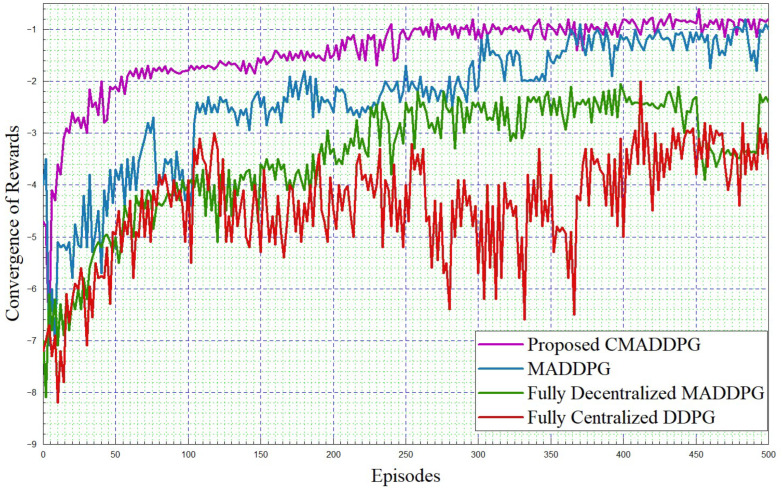
Convergence of rewards. P=8,V=5, intra-platoon spacing =5 m.

The simulation framework is shown in Figure 4. The performance comparisons of Reward function convergence for the benchmark algorithms and the proposed algorithm by varying the platoon numbers from seven to eight, and comprising five vehicles in each platoon, are plotted in Figure 5 and Figure 6, respectively. To begin with, it is evident that in the given T seconds duration, every agent (vehicle) can effectively accomplish the assigned task and attain the appropriate rewards. Further, it can be inferred that the reward functions (task-wise) converge under 50 episodes, eventually expediting the convergence time of the proposed CMADDPG algorithm and outperforming the other benchmark algorithms. Initially, exploring the fully centralized DDPG algorithm, it delivers deprived performance when compared with all other algorithms in both cases. The algorithm has to observe all the agents and decide on the action in a centralized manner, whereas the agents’ performance is not conceived explicitly, causing underperformance; the performance becomes worse as the number of agents escalates. Subsequently evaluating the next MADDPG algorithms, the decentralized behavior exacerbated the agents to be completely ignorant of the neighboring agent’s action and its corresponding policies, resulting in a higher interference and deteriorating the algorithm’s performance as a whole, as shown in Figure 5. Moreover, with the addition of platoons, the efficiency of the algorithm is aggravated more than that of centralized DDPG, as illustrated in Figure 6. It is apparent from the plot that the proposed CMADDPG method stabilizes quickly with few distortions while converging, primarily due to the effective cooperation of agents (vehicles) resulting in concurrent maximization of agents’ global and local rewards. In addition, the proposed framework is highly resistant to the errors generated by function approximations. The robustness is attained through proper evaluation of errors while updating the policies and values by global critics and also achieved through the exploration employed with Gaussian noise. Ultimately, the aforesaid crucial factors are also liable for the convergence of the proposed CMADDPG algorithm despite the densified vehicle population, as depicted in Figure 5 and Figure 6.

#### 4.2.2. Performance Comparison of Average Age of Information

**Figure 7:**
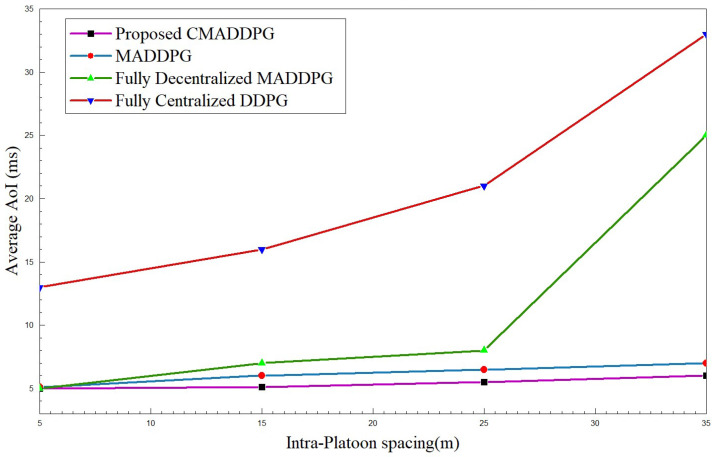
Average Age of Information vs. intra-platoon spacing for P=7,V=5.

**Figure 8:**
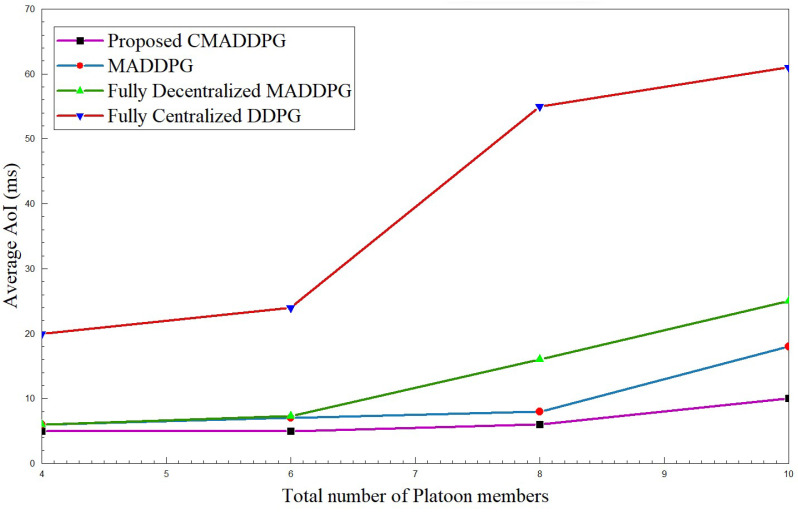
Average Age of Information vs. total number of platoon members for P=7, intra-platoon spacing =25 m.

The Average Age of Information of platoons against their intra-platoon spacing and the total number of platoon members is interpreted in Figure 7 and Figure 8. The time consumed for a successful transmission extends as the intra-platoon spacing widens for all the benchmark algorithms, as presented in Figure 7. As the spacing between the platoon broadens, the state of the channel between the Captain Vehicle and the Follower Vehicle fluctuates owing to the channel fading caused by the mobility of vehicles in the environment. Consequently, CV has to constantly focus on the intra-platoon mode of communication for retransmitting the CAM messages to the corresponding FVs, hence diffusing the focus on RSUs communication; thus, the average Age of Information intensifies. However, the proposed CMADDPG algorithm outperforms the benchmark algorithms by retaining a 5–10 ms average Age of Information span for P=7 and V=5 by also ensuring higher QoS. The MADDPG algorithm possesses similar behavior as the CMADDPG; yet, the proposed cascaded critics guarantee the AoI performance when the spacing between the platoons elongates above 25 m. In the time frame, as the platoon space widens to 30–35 m, the centralized DDPG and the decentralized MADDPG endure high interference to the neighboring platoons due to channel uncertainties and repeated transmission of CAM messages, illustrating a sudden leap in the plot, as shown in Figure 7 and Figure 8. The decentralized way of execution also deteriorates the effective resource allocation to platoon members causing interference in the environment as the members’ increases are conveyed as rapid changes in Figure 8.

#### 4.2.3. Performance Comparison of Transmission Probability of CAM Messages

**Figure 9:**
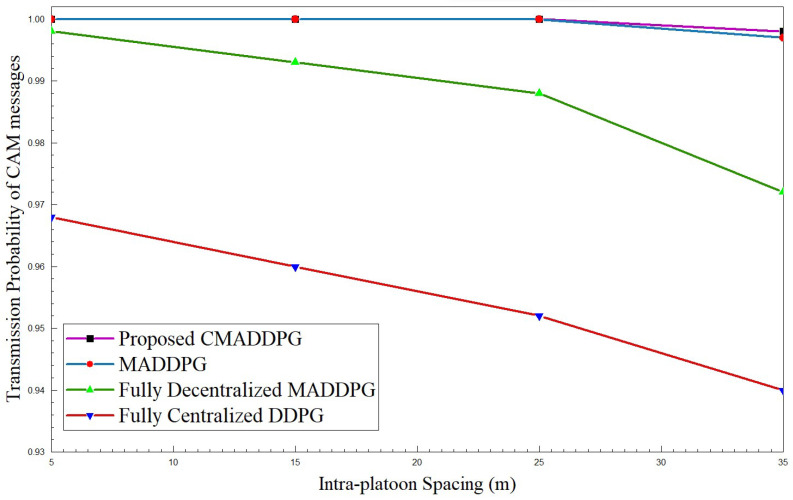
Transmission probability of CAM messages vs. intra-platoon spacing for P=7,V=5.

**Figure 10:**
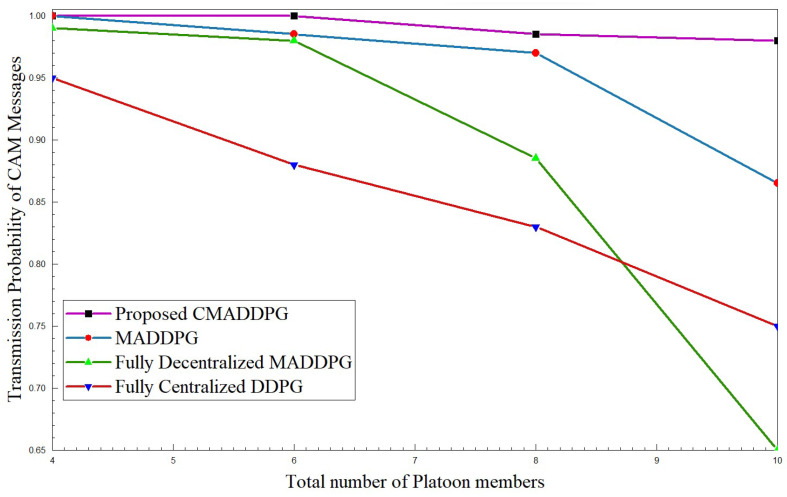
Transmission probability of CAM messages vs. number of platoon members for P=7, intra-platoon spacing =25 m.

Figure 9 and Figure 10 highlight the transmission probability of CAM messages with respect to intra-platoon spacing and the total number of platoon members. From the plot, it can be inferred that the probability of CAM message transmission tumbles with the broadening of intra-platoon space for the benchmark algorithms. Concerning the MADDPG and DDPG algorithms, the growth of vehicle population from 30 members to 50 in the environment highly influences the descent of transmission probability. At times, as the vehicle population densifies, the probability of decentralized MADDPG diminishes to 65 percent, which is inferior to Centralized DDPG, primarily due to deficits in interference management. The densification of the number of vehicles deteriorates the performance of transmission probability.

However, the proposed CMADDPG algorithms exhibit a reliable and robust nature towards variation in the spacing of platoons and the size of platoons. The transmission probability of CAM messages of the CMADDPG algorithm is retained at 99 percent even at 25 m intra-platoon spacing. Conversely, the transmission probability decrease is witnessed in the other benchmark algorithms for similar settings, as demonstrated in all the plots. It is evident that the proposed Cascaded Multi-Agent Deep Deterministic Policy Gradient significantly outperforms the other benchmark algorithms and demonstrates a resistant response against any fluctuations in the metric.

## 5. Conclusions

In this work, a CMADDPG-based resource management technique for an MEC-Aided platooning architecture is proposed and successfully implemented. The multi-objective optimization algorithm maximized the transmission reliability through two modes of operation. It also minimized the Age of Information metric while ensuring the transmission probability of CAM messages. The Captain Vehicles were designed to learn cooperatively with a desire to maximize both the global and local rewards. The MEC-Aided platooning helped in offloading the computational tasks, with an aim to minimize the overall latency of the platooning system. The cascaded critics hold accountability for balancing the estimation bias, where the effectiveness of the algorithm is clearly demonstrated. Regardless of the options provided to the CVs in independently choosing the communication mode and Resource Blocks, the proposed algorithm has proved its efficiency in resource management. The effectiveness of the mechanism is validated through in-depth simulations with a range of test scenarios of altered platoon sizes. In the future, the proposed approach is to be extended to heterogeneous vehicular network environments and its resource allocation schemes can be investigated.

## Figures and Tables

**Table 1:** List of primary notations and explanations.

Primary Notations	Explanation
V	group of positive integers
P/P/i	number/group/array of platoons
$V_{i}/V_{i}/n$	number/group/array of vehicles in platoon *i*
B/B/b	number/group/array of subchannels
$g_{i}\left[b\right]$	small-scale fading dependent frequency
$\alpha_{i}$	large-scale fading independent frequency
L	location of RSU
$\xi_{i,b}^{t}$	indicates the allocation of subchannel
$\theta_{i}^{t}$	indicates the selection of inter/intra-platoon mode
$p_{i}^{t}\left[b\right]$	power required by CV *i*
$C_{i,L}^{t}\left[b\right]$	data rate in the subchannel *b* linking the CV *i* and RSU
$C_{i,j}^{t}\left[b\right]$	data rate in the subchannel *b* linking the CV *i* and its FV
$h_{i,L}\left[b\right]$	channel gain in the subchannel *b* linking the CV *i* and RSU
$h_{i,j}\left[b\right]$	channel gain in the subchannel *b* linking the CV *i* and its FV
$AoI_{i}^{t}$	AoI of CV till the start of the scheduling window *t*
${ℵ}_{i}$	CV $i^{\prime }s$ CAM message size
$C_{i,L}^{min}$	CV’s least capacity requirement
E	collection of edge nodes
CT	the group of computing task
${\mathrm{Pp}}_{n}$	Processing power of edge nodes
$\phi_{i}^{n}\left(x\right)$	Boolean factor for discovering the vehicle location
*L*	total latency for executing CT
$es_{n},er_{n}$	caching space and computational resources required by edge nodes
$ct_{sn},cr_{rn}$	caching space and computational resources required by computation tasks

**Table 2:** Simulation parameters.

**Parameters–Vehicular Environment**	**Values**
Number of Resource blocks	3
Bandwidth of each Resource block	180 kHz
Carrier Frequency	2 GHz
Total number of vehicles	15–50
Platoon size	5–10
Intra-platoon spacing (in m)	5, 15, 25, 35
Speed of platoon	36–54 km/h
Mobility model of vehicles	Urban Case [9]
Power of vehicles	30 dBm
Noise power ($\sigma^{2}$)	−114 dBm
CAM Size	4 kB
CAM Transmission Time	10 ms
CV and RSU capacity requirement	3 bps/Hz
Fast Fading	Rayleigh fading
Channel information update	for every 10 ms
Edge node Tx power	46 dBm
Edge node Antenna gain	18 dB
Edge node blocks	20
**Parameters–Neural Network**	**Values**
Mini batch size	64
Experience Replay	50,000
Discount factor γ	0.99
Target network update parameter	0.0005
Hidden layers of local actor network	1024 and 512 neurons
Hidden layers of local critic network	512 and 256 neurons
Hidden layers of local actor network	1024, 512, and 256 neurons
Learning rate of critic/actor	0.001/0.0001
Episodes	500
Iterations for each episode	100

## Data Availability

Data are contained within the article.

## Abbreviations

The following abbreviations are used in this manuscript:

AI	Artificial Intelligence
AoI	Age of Information
AV	Automated Vehicle
BS	Base Station
BSM	Basic Safety Message
CACC	Cooperative Adaptive Cruise Control
CAM	Cooperative Awareness Messages
CC	Cloud Computing
C-UE	Cellular-User Equipment
CV	Captain Vehicle
D2D	Device-to-Device
D3PG	Deep Differentiable Deterministic Policy Gradient
DCF	Distributed Coordination Function
DRL	Deep Reinforcement Learning
DRR	Delivery Rate Ratio
DSRC	Dedicated Short-Range Communication
E2E	End-to-End
ETSI	European Telecommunications Standards Institute
FV	Follower Vehicle
Het-MEC	Heterogeneous Multi-access Edge Computing
ITS	Intelligent Transport System
LTE	Long-Term Evolution
MADDPG	Multi-Agent Deep Deterministic Policy Gradient
MARL	Multi-agent reinforcement learning
MEC	Multi-access Edge Computing
MOOP	Multi-Objective Optimization
NOMA	Non-Orthogonal Multiple Access
PaaS	Platform as a Service
QoS	Quality of Service
ReLU	Rectified Linear Activation Unit
RL	Reinforcement Learning
